# Testing the expectancy-disconfirmation theory: Geography, employment status and household size of local communities determine their perspectives of a local mine business in South Africa

**DOI:** 10.1371/journal.pone.0270815

**Published:** 2022-07-25

**Authors:** Mothusi Boihang, Kowiyou Yessoufou

**Affiliations:** Department of Environmental Management and Energy Studies, University of Johannesburg, Johannesburg, South Africa; Al Mansour University College-Baghdad-Iraq, IRAQ

## Abstract

Local communities’ perspectives on mining businesses are a matter of endless debate, particularly in developing countries. If misunderstood or mismanaged by authorities (local and national), these perspectives may lead to violent and deadly reactions, which are unaffordable given the tremendous contribution of mining businesses to socio-economic development. The recurrence of these violent events means that authorities and mining businesses may have been failing to understand the dynamic of local people’s expectations. Here, to explain the complexity of the interactions of local people with mine businesses, we collected socio-economic data along with data on people’s satisfaction levels towards the services delivered by a local mining business in the *Mose Kotane* Local Municipality in South Africa. Data collected were analyzed by fitting a Structural Equation Model (SEM). We found that only 4–8% of communities’ expectations were met by the local mine business, and that closest communities to the mine do not benefit significantly more services than away-communities (Chi-square = 2.71, df = 4, P = 0.60). However, the proportion of moderately satisfied people (in relation to the services delivered by the mine) tends to increase when moving away from the mine while the proportion of dissatisfied people decreases. Our SEM, linking socio-economic data to communities’ perspectives, shows a good fit (Fisher C value = 0; P = 1.00, n = 158). In communities away from the mine, residents who were initially happy about the establishment of the mining business tend to be satisfied with the services delivered by the mine (β = 2.69±0.41, P<0.001) but these residents are likely to be employed people (P<0.05). In communities close to the mine, large-sized households tend to be satisfied with the mine-delivered services (P = 0.04). This is potentially due to the fact that a large household is more likely to have at least one person working for the mine. Collectively, these findings reveal how socio-economic variables determine people’s perspectives on the mining business.

## 1. Introduction

Mining contributes significantly to the world economy and social development, bringing along employment opportunities, an influx of capital and economic development particularly in low- and middle-income regions [1, 2]. These mining regions include the Western, Southern and Central Africa, Oceania, Central Asia and Latin America [3]. Even in some developed countries, mining is also critical for economy. For example, on the list of top 50 countries ranked based on mining contributing index to economies, are found three high-income economies, as well as 17 upper middle-income economies, 16 lower-middle income economies and 14 low-income economies [3]. Furthermore, it is estimated that globally, mining contributes 1 to 3% to direct employment [4]. According to ref. [4], mineral production at the mine stage was valued at 300 billion USD in 1996 (roughly 0.6% of the world GDP). This value increased to 1800 billion USD (1.9% of global GDP) in 2011 and fell to 1200 billion USD in 2016 (1.2% of world GDP). As a result, the top 20 countries on the mining contributing index list improved their Human Development index by 27% [3]. Given the need for economic growth to meet the growing demands inherent to the rise of global population, it is expected that the demand for minerals will keep increasing in the future [5, 6].

In the meantime, two-thirds of close to 4 billion people living in the 50+ mining countries live in abject poverty [5], prompting the need to investigate how poor communities can collaborate, benefit, and uplift their socio-economic conditions due to proximities to profit-making mining companies. Recently corporate policies now make provision and explicitly address a range of broader social justice objectives, including employment of indigenous people, security and human rights, social impact assessment, ethical procurement, and stakeholder and/or community consultation [7, 8]. Interestingly, these polices do not only focus on mitigating negative impacts of mining on the environment and people, but also on delivering benefits for local, regional and even global communities [7]. Because historically the function of community relation was discredited in production-driven environments such as mining companies, not enough resources were allocated to that function. For example, a study done in Jordan by ref. [9] found that there haven’t been policies in place that force mining companies to contribute to the socio-economic well-being of local communities where they are mining. As such, the expectations and satisfaction of these communities could not be measured because there was no policy in place that forced the mining company to scope out the needs and expectations of the community [9]. It is therefore clear that these companies do not see the need to worry about whether local communities were satisfied with their activities and whether their expectations were met or not [7]. Instead, mining companies dealt with community issues in a reactive impromptu manner, rather than including them in their business strategies.

In Africa, mining is the largest single contributor to the economy of the Democratic Republic of Congo (DRC). For example, in DRC, mineral exports constitute 86% of total exports and mineral production value at the mine stage was 6.8 billion USD in 2016, representing 12% of GDP. In Tanzania, ref. [10] indicated that males are more likely to score a mining job than females and communities closer to the mine tend to have large households [10]. Communities closer to the mining site benefit more from employment, and the mine also contributes to improved road networks, water and school constructions [10]. In the 1970s, mining businesses in South Africa grew significantly to influence the economy and employment, and in the 1980s, the business became the second most influential industry contributing 21% to the country’s GDP [11]. Today, although mining businesses only contribute 8% to the country’s GDP, the mining businesses remain one of the most important employers, in term of its workforce [11]. However, mining activities may have, sometimes, adverse impacts on the socioeconomic wellbeing of local communities [12–14]. Unfortunately, these impacts are particularly felt heavily in small rural poor communities which are already vulnerable to any environmental change, owing to extreme poverty. They may even lead to violent protests, resulting for example, in what is known in South Africa as “the Marikana massacre” [15]. This makes it important to understand and account for the perspectives of local communities on mining businesses if we are to prevent frequent social unrests of local communities.

From this perspective, several studies have been conducted throughout South Africa (e.g., [12, 16]). Ref. [12] specifically aimed to understand the ongoing struggles between local poor communities and mining industries over land, mining revenues and public services in the *Sefikile* village, in the Moses Kotane Local Municipality (MKLM, North-West Province, South Africa). However, it remains unclear how deep is the gap between expected services by local communities versus actual services delivered to these communities by mining industries in this Municipality. It is also unclear whether communities that are geographically close to the mining operation sites benefit significantly more services (e.g., socio-economic infrastructure) from the mining businesses than communities that are far away. Our expectation is that geographically close communities would benefit more than far-away communities because they face more environmental consequences of mining operations than other communities due to their proximity to the operation sites. Also, if there should be some protests or vandalism activities, mining businesses are more vulnerable to close communities, and as such, we expect mining businesses to listen to these close communities as a matter of priority.

More importantly, we generally have a poor understanding not only of the complexity of the relationships between both entities (communities and mine business) but also of the determinants of communities’ perspectives, i.e., their satisfaction level towards the mining businesses. Peoples’ satisfaction level is predicted in the expectancy-disconfirmation theory. The theory suggests that peoples’ satisfaction about a given service is determined through comparison of the performance of the service delivered to them and their expectations from that service [17–19]. Through this comparison, three outcomes are possible, each of which determines the level of peoples’ satisfaction [18]: i) positive disconfirmation, when performance exceeds expectations, in which case people are delighted; ii) zero disconfirmation, when performance simply matches expectations, in which case peoples are satisfied with the service, and iii) negative disconfirmation when performance is lower than expectations, leading to people’s dissatisfaction or unhappiness.

The present study aims to test the expectancy-disconfirmation theory with the rationale that the outcomes could be used in the future to inform policy development towards socio-economic development and peaceful co-existence of mining industries and local communities. The rationale of the present study is that if the level of complexity and the satisfaction patterns of local communities are well understood, this could inform policy development to the benefits of both entities. For example, if the initial expectations of local communities from mining businesses are well understood as well as the determinants of these expectations at community rather than just country levels, this would facilitate clear monitoring and evaluation processes of service delivery by the mining businesses and thus help the prevention of tensed environment for both communities and mining industries.

To this end, three objectives are set: i) to document socio-economic services that local communities expect from the mining industry in the MKLM; ii) to identify socio-economic gap, if any, between expected and actually delivered services; and iii) to propose a framework or meta-model that explains the complexity of the relationships between socio-economic factors and communities’ perspectives on mining businesses. This meta-model is used to identify the determinants of communities’ perspectives on mining business.

## 2. Materials and method

### 2.1. Ethics

This paper is the product of the MSc dissertation in environmental management of the first author. The study was covered by the ethical approval from the Faculty Ethics Committee of the University of Johannesburg. No minors were targeted as respondents to our questionnaire, and all respondents gave their written consent prior to participating to the study. A sample of the consent form is provided as supplemental information.

### 2.2. Study site

This study took place in the North-West province of South Africa precisely in the Moses Kotane Local Municipality (MKLM) which is a category B4 municipality located within the *Bojanala* District Municipality. Category B4 Municipalities refer to municipalities located in rural areas, typically characterized by the presence of one or two small towns, communal land tenure and villages [20]. The MKLM covers an area of approximately 5220km² comprising 107 villages and two formal towns (*Mogwase* and *Madikwe*; [21]). Five of these villages, henceforth referred to as communities (*Legkraal*, *Lesetleng*, *Ramoga*, *Moruleng* and *Manamakgoteng*) are included in the present study, and they are located within the *Bakgatla-ba-kgafela* tribal area. The economy of MKLM is driven mainly by tourism, agriculture, and mining [21]. The MKLM hosts the *Pilanesburg Platinum Mine* which geologically sits on the western limb of the world famous bushveld igneous complex, the world’s largest platinum resources [22].

### 2.3. Data collection

**Fig 1:**
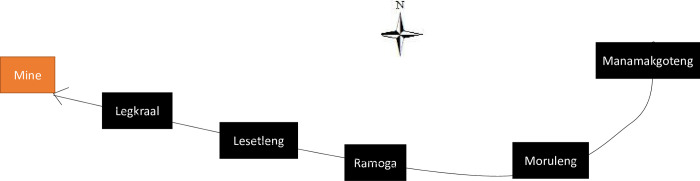
Spatial pattern of the positions of the five targeted villages for this study. In black are the positions of villages and in orange the position of the mine. The arrow indicates the direction from the furthest to the closest village to the mine location.

The selected research sites were five communities (*Legkraal*, *Lesetleng*, *Ramoga*, *Moruleng and Manamakgoteng*) in the North-West province, South Africa. These five communities were selected not only because of their proximity to the Plinaesburg Platinum Mine, but also because these villages are aligned, one after the other, in an increasing distance from the Plinaesburg Platinum Mine (Fig 1). Such alignment provides a unique setup that allows an investigation of how socio-demographic factors change along a distance gradient from the mining operation site (*Legkraal > Lesetleng > Ramoga > Moruleng > Manamakgoteng*). It also allows us to test whether services delivery by the local mining company to the community may have been influenced by the distance of the community from the mine location: our expectation is that geographically close communities would benefit more services from the mine than communities that are far away from the mine. The furthest community from the mining operation site is *Manamakgoteng* which is approximately 27km away from the mining operation site whereas *Legkraal* is the closest community to the site, 10Km from the mining operations.

Initially, a minimum of 30 households were planned to be selected randomly in each of the five communities. However, during data collection, some randomly selected households were empty. To get around this difficulty, the criteria of presence/absence of people in a household was then used to select a minimum of 30 households per village. This makes it a minimum of 150 households in the five communities where 158 people (respondents) were interviewed.

Data collection was conducted for a period of four weeks through semi-structured interviews. The interviews were designed in the form of a questionnaire to collect data on the following variables: people’s perspectives of the mining businesses, their expectations from the local mining industry, the actual services delivered by the mining industry and more importantly, their level of satisfaction *vis-à-vis* the delivered services. People’s perspectives were measured in two ways: i) happiness (are people happy when they first heard of the establishment of the mining business in the MKLM?) and ii) satisfaction status (are people satisfied of the services delivered by the mining business since establishment?). Satisfaction status was firstly measured as binary (satisfied vs. not satisfied) and secondly as a rank (dissatisfied, moderately satisfied, highly satisfied). Socio-demographic and economic data were also collected: gender, level of education, professional occupation, age, household size, distance of the respondent’s house from the mining site and residence time in the village. All raw data collected are presented in S1 Table (raw data) and S2 Table (coded data).

### 2.4. Data analysis

The first and second objectives of the study are to document expected and actual services delivered to local communities by the mining industry in the Municipality. This is a qualitative exercise where data were simply reported in a form of tables and graphs. However, for the last objective, all analyses were done in R [23], and all R scripts used for the analyses are provided in Supplemental Information (S1 Appendix).

Prior to this analysis, data collected were coded numerically as follows: Gender: Male = 1; Female = 0; Education level in number of years spent at school: 12 years = Grade 12; 14 years = Diploma/Certificate; 15 years = Degree/Postgraduate degree; Professional occupation: Unemployed = 0; Student = 1; Retired = 2; Employed = 3; Happiness: Happy = 1, Unhappy = 0; Satisfaction levels: Dissatisfied = 0, Moderately satisfied = 1; Highly satisfied = 2 (see S2 Table).

To understand the relationships between socio-economic variables and people’s perspectives, a number of expected relationships between variables were first defined and then translated into a structural equation model (SEM; see R script in S1 Appendix). This SEM was fitted to the data in S2 Table using the R function *psem* in the R library piecewiseSEM [24]. As showed in the R script, this SEM contains six GLM models (Generalized Linear Model) with a binomial error structure when the response variables are binary, e.g., happiness (happy vs. not happy). The adequacy of the SEM was tested based on its Goodness of fit (C value) and P value. An adequate SEM would show a low C value and a P > 0.05 [24, 25]. The explanatory power (R^2^) of each of the six GLM models included in the SEM analysis are also calculated.

## 3. Results

### 3.1. Socio-demographic characteristics of respondents

In total 158 respondents participated to this study from all five communities. The sociodemographic structure (S2 Table) shows that most respondents are 30 years old on average (mean age = 38±16.34). The household size in the community varies from 1 to 11 (mean size = 4.44±2.18). These communities are arranged along a distance gradient from 10km to 27km away from the mining operation site, with the *Ramoga* community found roughly at equal distance (18km) between the mining operation site and the furthest community to the mining site (*Manamakgoteng* community). In term of education levels, the vast majority of community members interviewed have matric level (i.e., high school certificate, 80%) with some having even tertiary education. Furthermore, community members interviewed have been residing in the study area for, on average, 31.82±18.12 years. Unfortunately, most of the community members are unemployed (42%) with a relatively close proportion of employed members (33%). Clearly, most people’s expectations concern infrastructure development or improvement, irrespective of their ages, socio-economic conditions, and geographic locations (S1 Table).

### 3.2. Socio-economic services delivered to the communities by the mine

**Fig 2:**
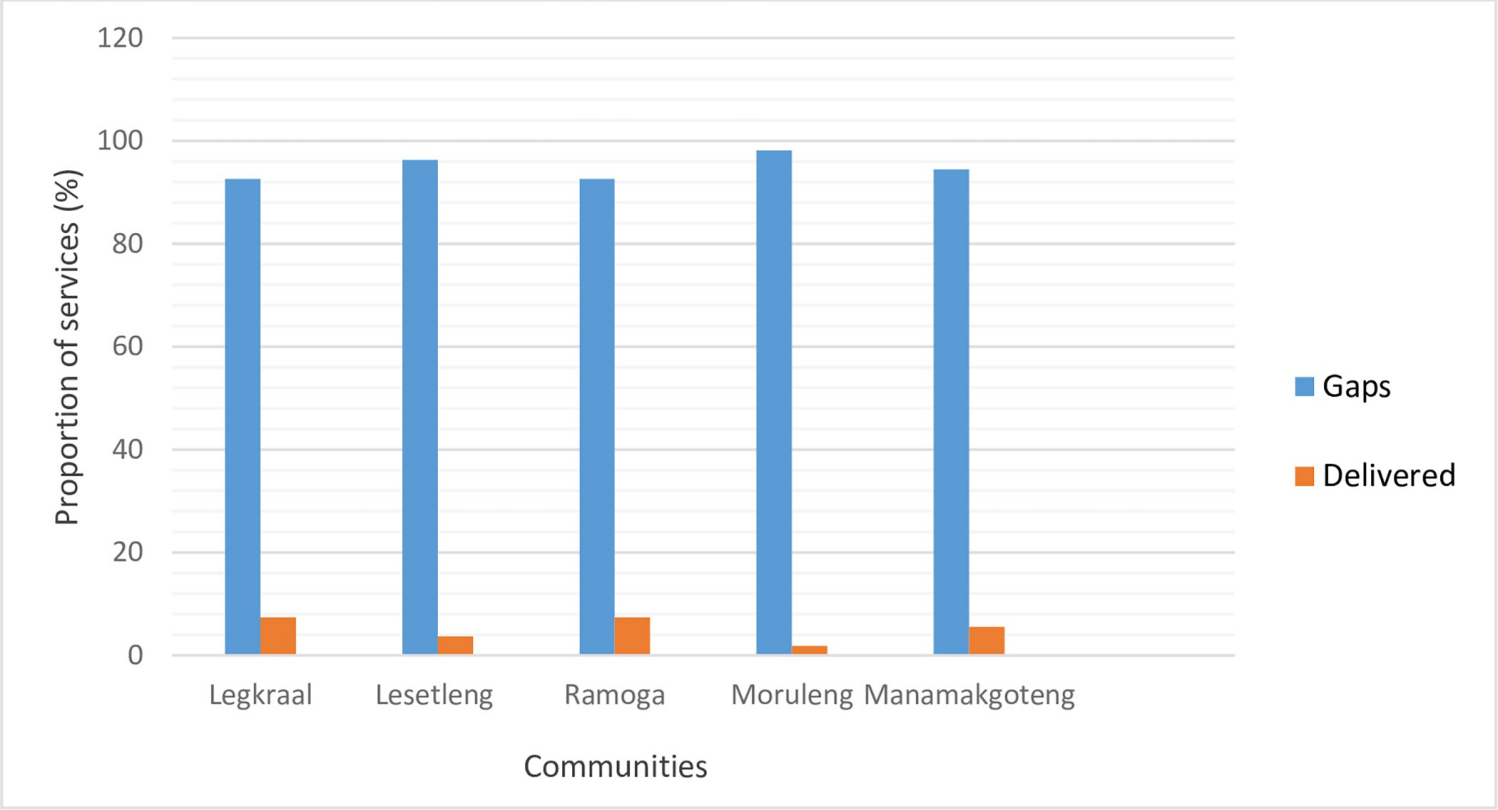
Comparative analysis of the proportion of services expected by the communities and the services actually delivered by the local mining industry. Blue bars = Proportion of expected services that are not delivered (Gaps); Orange bars = Proportion of services delivered by the local mining industry. Data used for this graph are in Table1. Communities are arranged from the closest to the furthest to the mining operation site.

**Fig 3:**
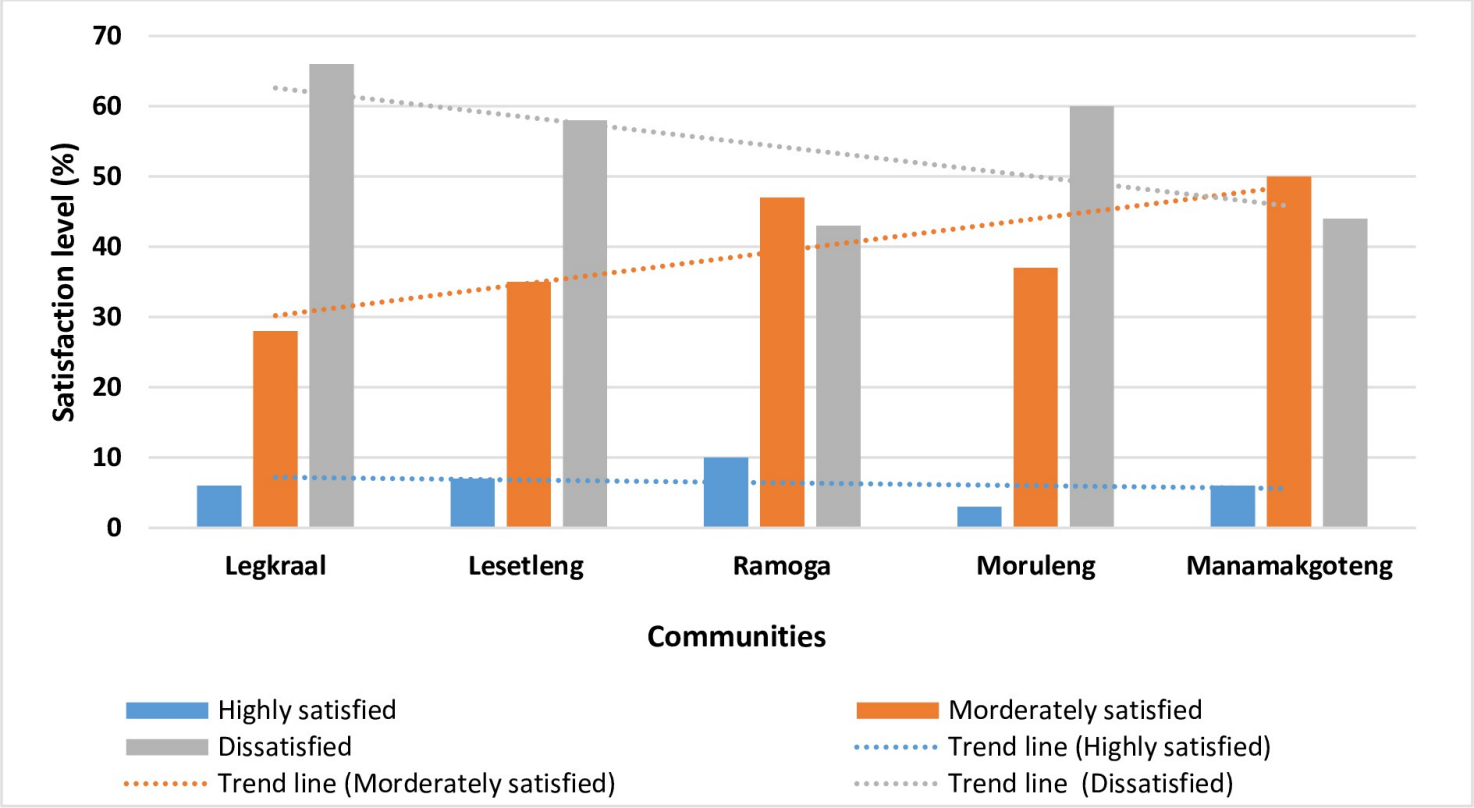
Patterns of satisfaction level of communities along a distance gradient (from the closest community to the furthest community away from the mining operation site).

All the socio-economic services that communities were expecting from the local mining company are provided in Table 1. These expected services are categorized into six (06) groups: Infrastructure development, Environmental Management, Educational development, Employment, Compensatory measures, and Security. A comparative analysis of expected versus delivered services shows that between 92% and 96% of expectations were not met while only 4–8% of expected services were delivered (Fig 2). Testing whether there were significant differences in the proportion of services actually delivered from the closest to the furthest community from the mining site, the analysis shows that there was no such difference along a distance gradient (chi-square = 2.71, df = 4, P = 0.60). Similarly, there seems not to be a clear pattern in the proportion of highly satisfied people as we move away from the mine unlike the proportion of moderately satisfied people which tends to increase while the proportion of dissatisfied people decreases along the same distance gradient (Fig 3).

**Table 1:** Expected and delivered services to local communities by the local mining industry. (-) = expected but not delivered services; (+) = exepected and actually delivered services; NA = services not reported as expected from the local mining industry in a given community.

Overall expectation		Legkraal	Lesetleng	Ramoga	Moruleng	Manamakgoteng
Infrastructure development	Hospital construction, the clinic only opens weekdays until 4pm. (the closest hospital is 38km)	-	-	-	-	-
	Renovation of old schools to hospitals	-	NA	-	NA	NA
	Roads construction	-	-	-	-	-
	Street lights construction	-	-	-	-	-
	Maintenance of existing street lights	-	NA	NA	-	NA
	Road drainage system construction	NA	-	-	NA	NA
	Introduction of mobile clinics to the area	NA	NA	-	-	-
	Construction of sports and recreational facilities	-	-	-	-	-
	Construction of gym facilities	-	NA	NA	NA	NA
	Construction of the community hall	-	-	+	-	-
	Road repairs	-	-	-	-	-
Environmental Management	Environmental protection and control of environmental impacts	-	NA	-	NA	NA
Services to communities	Bus service for school children	-	NA	-	-	-
	Construction of fencing for elderly people	NA	NA	-	-	-
	Housing construction and renovations for elderly people	-	NA	-	NA	NA
	Housing construction for mining employees	NA	NA	NA	-	-
	Free housing construction for local residents	NA	-	NA	-	-
	Water provision for livestock	-	NA	NA	NA	-
	Water provision for humans	+	-	-	-	-
	Construction of speed humps on the road which goes to the mine	-	NA	NA	NA	NA
	Electricity provision in clinics	-	NA	NA	NA	NA
	Electricity subsidies	-	-	-	NA	NA
	Medical supplies to clinics	NA	NA	-	NA	NA
	Financial assistance for the poor and childheaded families	NA	NA	-	-	-
	Construction of churches	NA	NA	NA	NA	-
	Maintenance of graveyards	-	-	-	-	-
Educational development	Construction of new schools	+	-	-	+	+
	Renovation of existing schools	-	-	+	-	+
	Construction of libraries	-	-	-	-	-
	Construction of skills development traning centre–cooking, baking, agriculture, computer	-	-	-	-	-
	Scholarships, internships and learnerships	-	-	-	-	-
	Construction of ABET (Adult Basic Education and Training) learning facilities	-	NA	NA	NA	NA
	Renovation of old schools to training centres	-	NA	-	-	-
	Funding of book clubs	NA	-	NA	NA	-
	Construction of a University	NA	-	NA	NA	NA
	Funding of schools to improve the curriculum at schools	NA	-	-	-	-
	Provision of food subsidies to schools	NA	-	-	-	-
	Suppy internet to schools	NA	NA	NA	-	-
Employment	Employment of local people	-	+	+	-	-
	Create jobs for the youth	+	-	+	-	-
	Create non-mining related jobs	-	NA	-	-	NA
	60% of the mine work force should include people living within a 50Kms radius from the mine	NA	NA	-	-	NA
	People, older than 30 years, should also be considered for employment	NA	NA	-	NA	NA
	There should be transaprency between the mine and the local community	-	NA	NA	NA	NA
	Sub-contract projects form the mine to local people	-	-	NA	-	NA
	Improve the mining recruitment process	-	NA	NA	-	NA
	Mine should empower local businesses	NA	+	NA	-	-
	Procument should not only include catering (construction and logistics)	NA	-	NA	-	-
Compensatory measures	Monetary compensation to the community every two years	-	-	NA	-	-
	Compensation for landowners (cattle and crop farmers)	-	NA	NA	NA	NA
	Repairing of cracked houses	+	-	-	NA	NA
Security	Police station construction	NA	NA	NA	NA	-

### 3.3. The complex relationships between socio-economic factors and peoples’ perspectives on mine

#### 3.3.1. Fitness of the SEM model and significant relationships

**Fig 4:**
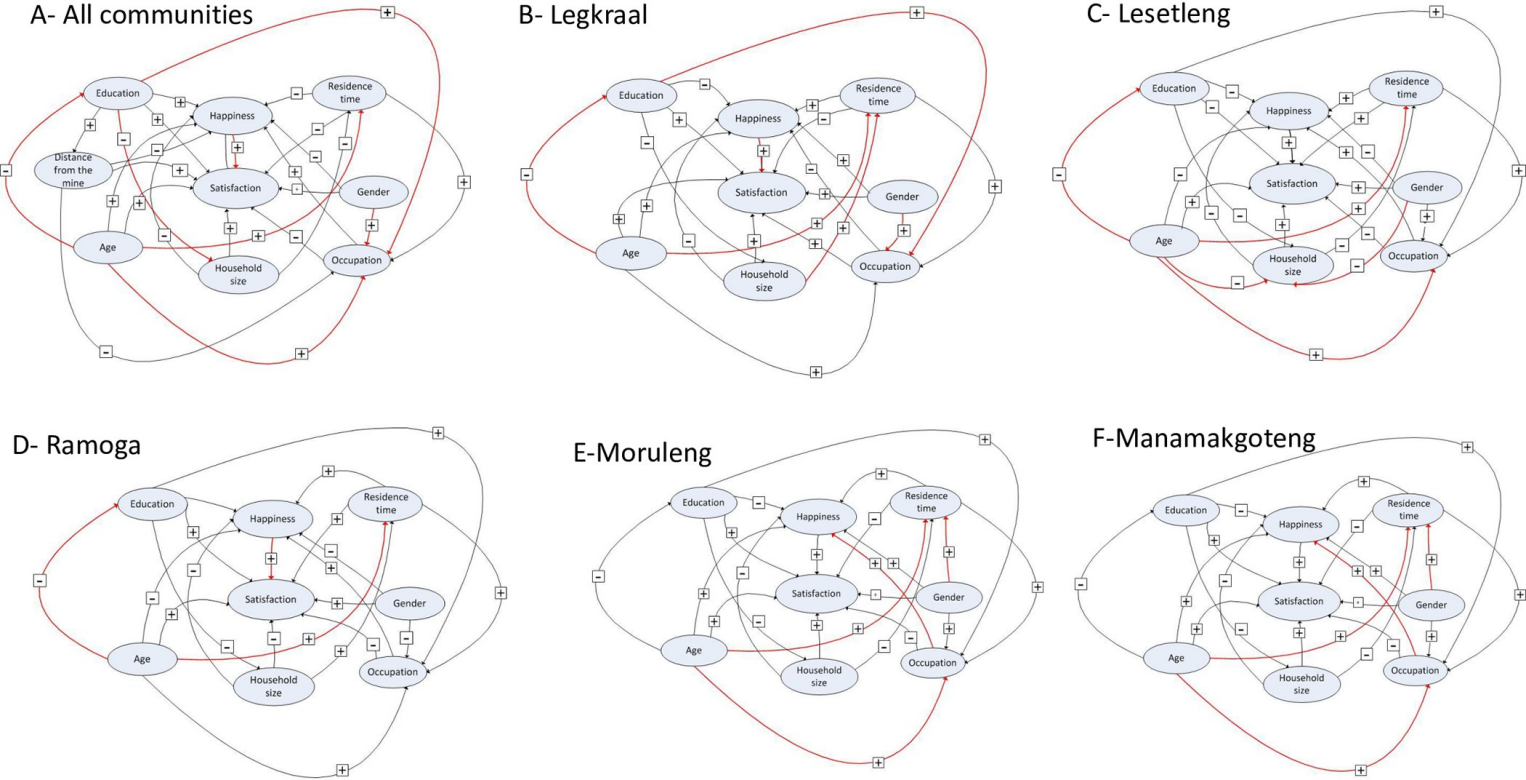
Structural equation model (SEM) illustrating the complex relationships between socio-demographic factors and people’s perspectives on mining business. “Happiness” is the variable used to assess whether people were initially happy when they heard for the first time about the establishment of a mining business in their communities; “Satisfaction” stands for whether people are satisfied or not of the services delivered by the mining business. “Occupation” stands for professional occupation (people are employed or not). A- When data from all five communities are combined; B- Legkraal community, C- Lesetleng community, D-Ramoga, EMoruleng, F- Manamakgoteng.

The proposed SEM model linking socio-economic and demographic factors to peoples’ perspectives on mine, showed in Fig 4A, has an appropriate goodness of fit to the data collected (Fisher C value = 0; P = 1.00, n = 158). In this SEM, there are significant relationships between some socio-demographic variables and the distance from the mining operation site (Table 2): larger households tend to be found further away from the mine (β = 1.08±0.22, P<0.001) and community members who have been residing for longer period in the MKLM are also found further away from the mine, but this relationship is only marginally significant (β = 0.04±0.02, P = 0.079). The SEM further reveals a clear pattern of gender and age inequality in employment status in the study area. Specifically, there is a positive correlation between gender and employment status such that males are more likely to be employed compared to females (β = 0.39±0.14, P = 0.005, Table 2). There is also a positive significant correlation between education levels and employment status (β = 0.08±0.02, P = 0.005, Table 2), such that highly educated people are more likely to be employed. However, the analysis showed a negative correlation between age and education level (β = -0.06±0.01, P<0.001; Table 2), and this indicates that older people tend to be less educated than the youth.

**Table 2:** Path coefficients of all relationships among variables included in the SEM model for all communities combined.

	Response	Predictor	Estimate	Std.Error	DF	Crit.Value	P.Value
1.	Happiness	Level of education	0.0072	0.0708	150	0.1022	0.9186
2.	Happiness	Residence time	-0.0090	0.0147	150	-0.6160	0.5415
3.	Happiness	Gender	-03151	0.3546	150	-0.8886	0.3742
4.	Happiness	Professional occupation	0.1132	0.1931	150	0.5862	0.5578
5.	Happiness	Age	0.0015	0.0173	150	0.0865	0.9311
6.	Happiness	Household size	-0.393	0.0828	150	-0.4745	0.6351
7.	Happiness	Distance form mine	0.0145	0.0274	150	0.5267	0.5984
8.	Satisfaction level	Level of education	0.0278	0.0870	149	0.3194	0.7495
9.	Satisfaction level	Residence time	-0.0155	0.0182	149	-0.8518	0.3943
10.	Satisfaction level	Gender	0.4876	0.4423	149	1.1025	0.2702
11.	Satisfaction level	Happiness	2.6938	0.4112	149	6.5506	0.0000
12.	Satisfaction level	Professional occupation	-0.0523	0.2360	149	-0.2216	0.8246
13.	Satisfaction level	Household size	0.0994	0.1032	149	0.9634	0.3354
14.	Satisfaction level	Age	0.0149	0.0215	149	0.6925	0.3354
15.	Satisfaction level	Distance from mine	0.0437	0.0340	149	1.2848	0.1989
16.	Household size	Level of education	-0.0358	0.0157	154	-2.2792	0.0227
17.	Household size	Gender	-0.2097	0.0771	154	-2.7206	0.0065
18.	Household size	Age	-0.0066	0.0026	154	-2.5302	0.0114
19.	Distance from mine	Level of education	0.0784	0.1962	154	0.3994	0.6901
20.	Distance from mine	Household size	1.0840	0.2236	154	4.8478	0.0000
21.	Distance from mine	Residence time	0.0495	0.0280	154	1.7660	0.0794
22.	Level of education	Age	-0.0608	0.0118	155	-5.1592	0.0000
23.	Level of education	Gender	-0.4244	0.3933	155	-1.0709	0.2823
24.	Residence time	Household size	-0.0166	0.4224	154	-0.0394	0.9686
25.	Residence time	Age	0.8787	0.0558	154	15.7557	0.0000
26.	Residence time	Gender	4.2170	1.8789	154	2.2444	0.0262
27.	Professional occupation	Level of education	0.0812	0.0285	152	2.8466	0.0050
28.	Professional occupation	Residence time	0.0024	0.0062	152	0.3887	0.6980
29.	Professional occupation	Gender	0.3997	0.1429	152	2.7965	0.0058
30	Professional occupation	Age	0.0197	0.0071	152	2.7755	0.0062
31.	Professional occupation	Distance from mine	-0.0161	0.0107	152	-1.4966	0.1366

Furthermore, age is a positive significant predictor of employment status (β = 0.01±0.007, P = 0.006, Table 2), suggesting that the youth is less likely to be employed in the region. Furthermore, the size of households in the community are also dependent on age, education levels and gender such that age (β = -0.006±0.002, P = 0.01, Table 2) and education level (β = -0.003±0.001, P = 0.002, Table 2) correlate negatively with household size whereas males tend to live in smaller households than females do (β = - 0.035±0.015, P = 0.0227, Table 2), although the former has been living in the area longer than the latter (β = 4.21±1.87, P = 0.02, Table 2).

#### 3.3.2. Determinants of people’s perspectives on the mining industry

People’s perspectives were measured as *happiness* (meaning: are people happy about the establishment of the mining industry in their locality?) and *satisfaction level* (are people satisfied of the services delivered by the mining industry?). Among all the variables tested, only happiness correlates significantly and positively with satisfaction level, indicating that residents who were happy about the establishment of the mining industry in their community tend to be satisfied with the services delivered by the mine to their community (β = 2.69±0.41, P<0.001, Fig 4A, Table 2). However, when moving away from the mine, the analysis shows some specificities (Fig 4B–4F; S3–S6 Tables). In the *Legkraal*, *Ramoga* and *Manamakgoteng* communities, people who are happy about the establishment of the mine tend to be satisfied with the services provided by the mine: *Legkraal* (β = 2.43±1.15, P = 0.03, Fig 4B, S3 Table), *Ramoga* (β = 2.69±1.10, P = 0.014, Fig 4D, S4 Table) and *Manamakgoteng* (β = 2.33±1.05, P = 0.02, Fig 4F, S5 Table). In addition, and perhaps surprisingly, large-sized households in the *Legkraal* tend to be satisfied with the mining business (β = 0.67±0.33, P = 0.04, Fig 4B, S3 Table). However, there was no such relationships in the remaining two communities: *Lesetleng* (β = 75.56±19192.50, P = 0.99, Fig 4C, S6 Table) and *Moruleng* (β = 872.3±322723.9, P = 0.99, Fig 4E, S7 Table). Instead, in the *Moruleng* and *Lesetleng* communities, happiness is predicted by professional occupation (β = 1.505±0.76, P = 0.04,) and gender (β = -2.15±1.11, P = 0.05).

## 4. Discussion

### 4.1. Mismatch between expectations and services delivered by the local mining Industry

In the MKLM, communities’ expectations from the local mining company are centered around infrastructure development (hospital, schools, roads, streetlights, recreational facilities, etc.), environmental management, socio-services (transport for school learners, houses, water, electricity and financial aid), educational development (school constructions, internships and scholarships, funding for schools), employment (employment of locals), compensatory measures, security and better living standards. Although these expectations are also reported in several other studies [9–14, 26, 27], they are generally qualified by mining businesses as unrealistic [14]. Because mining activities pollute the environment, local communities believe that they are entitled to a share in the companies’ profits as they have to bear the brunt of negative impacts from mining [27, 28]. Several studies reported that communities’ expectations are influenced by various factors, including their socio-economic conditions and government practices, their perceptions of impacts of mining and resource ownership, and the short-term nature of mining [26, 29–32]. These factors are also likely to determine communities’ perspectives of mining industries in the MKLM. We would have expected these factors to be determinant in people’s types of expectations from the mine in the MKLM. For example, younger people might have higher expectations of services delivered, or unemployed people might pay more attention to infrastructure development or educational development, thus making them more rigorous or demanding in how services are delivered. However, our data suggests that similar expectations cut across all ages and socio-economic groups focusing primarily on infrastructure development or improvement. The similarity in people’s expectations implies that similar socio-economic in the area are shared by everyone across demographic groups. If everyone, irrespective of socio-economic groups shared similar expectations, this means that problems raised in their expectations must be real and therefore deserve attention from decision makers.

Interestingly, the local mining company did provide some services to communities (e.g., high school, crèche, etc.) but these services represent only 4–8% of the communities’ expectations, implying that more need to be done in term of meeting residents expectations. This matches the negative disconfirmation scenario of the expectancy-disconfirmation theory [17–19, 33]. Obviously, the mining company, as a profit-making company, cannot do everything for the community, especially cannot deliver services that typically fall into the discretion of local authorities, e.g., building malls or ensuring security to residents, etc. Ref. [28] identified the root causes of the disparities between people’s expectations and actually delivered services by mining companies as poor communication, broken promises and a lack of working agreements between the mines and local people.

A consequence of the mismatch between communities’ expectations and the services actually delivered by mining companies is social tensions or social unrests [28, 34]. For example, in the study area, civil unrest broke out in March 2018 when local residents embarked on a service delivery protest in the *Manamakgoteng* (one of the communities included in the present study) and *Segakwaneng* communities. Community members argued that the mining company uses their road on a daily basis but yet, it does not fix the road or construct new ones. This is evidence that unfulfilled promises by mining companies lead to social tension that may turn deadly in some cases, e.g., the Maricana massacre in South Africa [15, 35–37].

### 4.2. Are local communities satisfied with the services delivered?

The scenario of negative disconfirmation we reported above implies that peoples’ expectations from the mining company are very high such that we should expect high level of dissatisfaction [38, 39] towards service delivery by the mine. However, based on the finding that closer communities to the mining site benefits more from the mine [10], our expectation was that communities close to mining operation sites would benefit significantly more services from the mining industry than communities far away. Unfortunately, this expectation does not hold in the MKLM, suggesting that there were no differences in the proportion of services delivered across all five communities. This is in contrast to what ref. [28] reported in the Peruvian town of Cajomerca in South America where the closest community to mine benefited more from job creation and an investment worth US$7 million in roads, schools, and local infrastructure. In the MKLM, the results show that, from the closest community to the mining site (Legkraal) to the furthest community (Manamakgoteng), the proportion of highly satisfied people does not show any apparent trend. This is not surprising, given that only 4–8% of the overall expected services by the communities are delivered by the mining company (negative disconfirmation).

This lack of prioritization of close communities for service delivery could also be because, in general, most mining businesses do not conduct appropriate stakeholder engagements with local communities [40] in such a way that their expectations are well understood. However, the proportion of moderately satisfied people tends to increase when moving away from the mine. This is understandable because people living far away from the mining site would not feel the negative effects of mining operations as severely as people living close to the mine (pollution, cracked houses, etc., [26, 41]). This is actually the reason why the proportion of dissatisfied people decreases when moving away from the mine. Our results also indicate that people living far away from the mine are not only happy about the establishment of the mining business in the MKLM but also satisfied with the services so far delivered by the mine. These people are mostly employed people because, far away from the mine, alternative job opportunities do exist, and residents do not rely exclusively on the mine, unlike communities close to the mine.

At community level, in the Legkraal community, the closest community to the mining site, there is a positive correlation between gender and professional occupations, implying that males are more likely to be employed than females, and highly educated people tend to be employed. Because these two significant relationships are not found in any other community as we move away from the mining operation site, this may imply two possible scenarios: either the mining industry employed preferentially males of the closest community (Legkraal) or it is the males employed by the mine who prefer to live closer to the mine in Legkraal. However, this study did not specifically distinguish between people working in the mine from those working elsewhere; it therefore remains possible that the employment status of the respondents interviewed may not always be linked to the presence of the mining company.

Three significant relationships are found in the Lesetleng community, the second closest community to the mining operation site and where the traditional Chief of all communities resides: age correlates negatively with household size (i.e., young people tend to live in large household) but positively with professional occupation (old people are more likely to be employed) whereas gender correlates negatively with household size, i.e., females live in large household. The Ramoga community is unique among all in that, apart from the common relationships between age and education level vs residence time reported above for all communities, there is no other significant relationship among the socio-demographic parameters that are specific to the Ramoga community. It is in this community that most of local residents employed in the mining industry originate from. This community is one of the youngest communities in terms of the residence time of the respondents. The Ramoga is close to the Moruleng community and therefore has access to the services and infrastructure provided in the Moruleng community. The Ramoga community is one of the only communities which have benefited significantly from the mining company with a community hall and a *crèche* built by the mine. Most of the respondents who participated in this study and who work in the local mining industry are from the *Ramoga* community.

In the second furthest community, i.e., the *Moruleng* community, which is considered as the capital city of the Moses Kotane Local Municipality, are found most of the socioeconomic infrastructure (mall, banks, post office, petrol station, the local soccer stadium, tribal offices, etc.) of the municipality. In this community, professional occupation and happiness (about the establishment of the mine business) correlate positively, implying that people who are already employed tend to be happy about the establishment of the mining business in the MKLM. This is surprising *a priori* simply because the first logical expectation would be that unemployed people should be those who should be happy that a mining business is coming into their community, given the job opportunity that the mining might provide. This expectation is true only in the *Manamakgoteng* community, the furthest community from the mine. People in the *Moruleng* community do not concern much themselves with mining related business simply because most of the community members involved in this study are employed in sectors other than the mine. Some of them take part in informal businesses on the side of the road.

Overall, people’s perspectives about the mine (i.e., are they happy or not of the establishment of the mining company in the MKLM) become apparent in the two furthest communities from the mine (*Moruleng* and *Manamakgoteng*) as it is only in these communities that significant predictor of happiness was found. The furthest community from the mining operation site is the *Manamakgoteng* community. The *Manamakgoteng* community is closer to *Moruleng*. This community is not as remote as *Legkraal* and *Lesetleng*: the transport system, infrastructure and services are easily accessible. The *Manamakgoteng* community also hosts larger households compared to the other four communities. There are two significant relationships identified in the *Manamakgoteng* community. Community members who work are generally happy of the establishment of the mine.

### 4.3. Determinants of communities’ perspectives

At the MKLM level, our SEM model indicates significant relationships between some socio-demographic variables and the distance from the mining operation site: larger households tend to be found further away from the mine, and community members who have been residing for longer period in the MKLM are also found further away from the mine. Interestingly, there is a positive relationship between happiness and satisfaction level, indicating that residents who were happy about the establishment of the mining business in their communities tend to be satisfied with the services delivered by the mine. Given that the mine delivered very less than expected (see also [26, 42], the relationship between happiness and satisfaction level could be purely cognitive (i.e., someone is happy that a mine company is coming to his community and is consequently also satisfied with what the company has achieved, no matter what).

At community level, the determinant of satisfaction level in the *Legkraal*, *Ramoga* and *Manamakgoteng* communities, is also the same as at municipality level, which is that people who are happy about the establishment of the mine tend to be satisfied with the services provided by the mine. However, and perhaps surprisingly, it is household size that determines the satisfaction level in the *Legkraal* community such that large-sized households tend to be satisfied with the mining business. Nonetheless, there was no such relationships in any other communities. Instead, in the *Moruleng* and *Lesetleng* communities, happiness about the mine is predicted by professional occupation (positively) and gender. Overall, people’s perspectives (happiness and satisfaction level) are determined by distance (how far they live from the mine), employment status and gender.

## 5. Conclusion

The present study took place in the MKLM in the North-West province of South Africa. Based on our sample of residents, residents of the MKLM are on average 38 years old, of which 80% have at least a matric level (high school certificate) and 42% are unemployed. We set three objectives for this study. The first one was to document people’s expectations from a local mining business operating in the area. We found six categories of expectations comprising Infrastructure development, Environmental Management, Educational development, Employment, Compensatory measures, and Security. Our second objective was to compare the expectations to the delivered services. We found that only 4–8% of expected services were delivered. Contrary to our expectation, the proportion of delivered services does not change significantly from the closest to the furthest community to the mine. However, we found that people’s satisfaction levels depend on the distance of communities from to the mine location. This is already an important variable (distance) to factor in policy development towards improving the relationships between residents and mining operations.

Lastly, we investigated the determinants of people’s perspectives on the local mining business. We built several models showing these perspectives measured as happiness and satisfaction levels. Our models show that peoples perspectives depend on their geographic locations. For example, in *Legkraal*, *Ramoga* and *Manamakgoteng*, our models indicate that people’s satisfaction depends on their initial mindset about the mining, that is, people who were initially happy about the establishment of the mining business in their areas are satisfied of the services delivered by the latter to the communities. However, the size of household is another important determining factor of residents’ satisfaction but only in *Legkraal* whereas professional occupation and gender determine whether people are happy or not about the mining business in *Moruleng* and *and Lesetleng*.

Our study therefore indicates how complex are the relationships between socioeconomic, demographic, communities’ geographic locations and their perspectives on mining businesses. Such complexity needs to be understood and factored in policy development for the benefits of both communities and mining businesses. This is important since it will reduce random decisions or only politically motivated decisions by the mining businesses. Furthermore, there is a need to reduce the disparity between community-based expectations and actually delivered services by the mine through effective regular communication channel between both parties. Such channels could include public meetings in which locals can express their concerns and provide input to various development phases of the mine [28]. This is important for the buy-in of local communities into mining operations and strategic decisions for peaceful collaborations. This is possible as revealed in a recent study where ref. [3] compared the level at which mining companies in Sweden and South Africa met the expectations of the local people. We suggest that closest communities to the mine should be prioritized in development activities since they are the ones heavily affected by environmental and social issues linked to mining. Once satisfaction level is high in closest communities, these communities become natural defenders of the mining operations against any external attacks, thus securing peaceful collaborations between communities and businesses. Finally, the expectancy-disconfirmation theory of satisfaction should be included in monitoring programme of the dynamic of the relationships between mining company and local communities.

## Supporting information

S1 Table Raw data collected from the field. (XLS)

S2 Table Coded data analyzed. (DOC)

S3 Table Path coefficients for all relationships among variables included in the SEM model for the Legkraal community. (DOC)

S4 Table Path coefficients for all relationships among variables included in the SEM model for the Lesetleng community. (DOC)

S5 Table Path coefficients for all relationships among variables included in the SEM model for the Ramoga community. (DOC)

S6 Table Path coefficients for all relationships among variables included in the SEM model for the Moruleng community. (DOC)

S7 Table Path coefficients for all relationships among variables included in the SEM model for the Manamakgoteng community. (DOC)

S1 Appendix R script used for data analysis. (R)
