# Supplementary material for: Testing the expectancy-disconfirmation theory: Geography, employment status and household size of local communities determine their perspectives of a local mine business in South Africa
Source: PLoS One. 2022 Jul 25;17(7):e0270815. doi: 10.1371/journal.pone.0270815 (PMC9312416; doi:10.1371/journal.pone.0270815)
Supplement: S2 Table — (DOC) [file pone.0270815.s002.doc]

**S2 table**. All data collected and analysed in the present study. Highlighted columns correspond to the metrics used for people’s perspectives. For data analysis purpose, data collected were coded as follows: Gender: Male = 1; Female = 0 - Education level in years: 12 years = Grade 12; 14 years = Diploma/Certificate; 15 years = Degree/Postgraduate degree - Professional occupation: Unemployed = 0; Student = 1; Retired = 2; Employed = 3 - Happiness: Happy = 1, Unhappy = 0 - Satisfaction levels: Dissatisfied = 0, Moderately satisfied = 0; Highly satisfied = 1.
