# Supplementary material for: Testing the expectancy-disconfirmation theory: Geography, employment status and household size of local communities determine their perspectives of a local mine business in South Africa
Source: PLoS One. 2022 Jul 25;17(7):e0270815. doi: 10.1371/journal.pone.0270815 (PMC9312416; doi:10.1371/journal.pone.0270815)
Supplement: S3 Table — (DOC) [file pone.0270815.s003.doc]

**S3 Table:** Path coefficients for all relationships among variables included in the SEM model for the *Legkraal* community.

|  | Response | Predictor | Estimate | Std.Error | DF | Crit.Value | P.Value |
| --- | --- | --- | --- | --- | --- | --- | --- |
| 1 | Happiness | Level of education | -0.1898 | 0.2042 | 25 | -0.9293 | 0.3528 |
| 2 | Happiness | Residence time | 0.0383 | 0.0337 | 25 | 1.1366 | 0.2557 |
| 3 | Happiness | Gender | 0.6213 | 1.0858 | 25 | 0.5722 | 0.5672 |
| 4 | Happiness | Professional occupation | -0.0822 | 0.5846 | 25 | -0.1406 | 0.8882 |
| 5 | Happiness | Age | -0.0344 | 0.0479 | 25 | -0.7188 | 0.4723 |
| 6 | Happiness | Household size | -0.1340 | 0.2701 | 25 | -0.4961 | 0.6198 |
| 7 | Satisfation level | Level of education | 0.3565 | 0.2841 | 24 | 1.2551 | 0.2095 |
| 8 | Satisfation level | Residence time | 0.0048 | 0.0406 | 24 | 0.1177 | 0.9063 |
| 9 | Satisfation level | Gender | 0.0449 | 1.2032 | 24 | 0.0373 | 0.9702 |
| 10 | Satisfation level | Happiness | 2.4394 | 1.1500 | 24 | 2.1213 | 0.0339 |
| 11 | Satisfation level | Professional occupation | 0.2984 | 0.6490 | 24 | 0.4598 | 0.6457 |
| 12 | Satisfation level | Household size | 0.6700 | 0.3318 | 24 | 2.0193 | 0.0435 |
| 13 | Satisfation level | Age | 0.0228 | 0.0525 | 24 | 0.4345 | 0.6639 |
| 14 | Household size | Level of education | -0.0060 | 0.0481 | 28 | -0.1250 | 0.9005 |
| 15 | Household size | Gender | 0.1425 | 0.2120 | 28 | 0.6723 | 0.5014 |
| 16 | Household size | Age | -0.0123 | 0.0079 | 28 | -1.5636 | 0.1179 |
| 17 | Level of education | Age | -0.0749 | 0.0271 | 29 | -2.7608 | 0.0099 |
| 18 | Level of education | Gender | -0.5775 | 0.8440 | 29 | -0.6842 | 0.4993 |
| 19 | Residence time | Household size | 0.1853 | 1.6262 | 28 | 0.1140 | 0.9101 |
| 20 | Residence time | Age | 0.9291 | 0.1701 | 28 | 5.4631 | 0.0000 |
| 21 | Residence time | Gender | 0.1313 | 5.0296 | 28 | 0.0261 | 0.9794 |
| 22 | Professional occupation | Level of education | 0.1393 | 0.0609 | 27 | 2.2851 | 0.0304 |
| 23 | Professional occupation | Residence time | 0.0059 | 0.0105 | 27 | 0.5588 | 0.5809 |
| 24 | Professional occupation | Gender | 1.1990 | 0.2670 | 27 | 4.4913 | 0.0001 |
| 25 | Professional occupation | Age | 0.0107 | 0.0146 | 27 | 0.7320 | 0.4705 |
