# Supplementary material for: Testing the expectancy-disconfirmation theory: Geography, employment status and household size of local communities determine their perspectives of a local mine business in South Africa
Source: PLoS One. 2022 Jul 25;17(7):e0270815. doi: 10.1371/journal.pone.0270815 (PMC9312416; doi:10.1371/journal.pone.0270815)
Supplement: S4 Table — (DOC) [file pone.0270815.s004.doc]

**S4 Table:** Path coefficients for all relationships among variables included in the SEM model for the *Lesetleng* community.

|  | Response | Predictor | Estimate | Std.Error | DF | Crit.Value | P.Value |
| --- | --- | --- | --- | --- | --- | --- | --- |
| 1 | Happiness | Level of education | -0.2672 | 0.2963 | 25 | -0.9020 | 0.3671 |
| 2 | Happiness | Residence time | 0.0368 | 0.0428 | 25 | 0.8597 | 0.3899 |
| 3 | Happiness | Gender | -2.1516 | 1.1186 | 25 | -1.9235 | 0.0544 |
| 4 | Happiness | Professional occupation | 1.0296 | 0.7011 | 25 | 1.468 | 0.1420 |
| 5 | Happiness | Age | -0.0578 | 0.0460 | 25 | -1.2568 | 0.2088 |
| 6 | Happiness | Household size | -0.1399 | 0.2392 | 25 | -0.5849 | 0.5586 |
| 7 | Satisfation level | Level of education | -0.3643 | 0.6109 | 24 | -0.5963 | 0.5510 |
| 8 | Satisfation level | Residence time | 0.9357 | 314.2779 | 24 | -0.0030 | 0.9976 |
| 9 | Satisfation level | Gender | 48.6730 | 22865.3089 | 24 | 0.0021 | 0.9983 |
| 10 | Satisfation level | Happiness | 75.5699 | 19192.5043 | 24 | 0.0039 | 0.9969 |
| 11 | Satisfation level | Professional occupation | -24.6407 | 7458.9743 | 24 | -0.0033 | 0.9974 |
| 12 | Satisfation level | Household size | 0.5092 | 0.8255 | 24 | 0.6168 | 0.5373 |
| 13 | Satisfation level | Age | 0.8692 | 314.2780 | 24 | 0.0028 | 0.9978 |
| 14 | Household size | Level of education | -0.0887 | 0.0508 | 28 | -1.7463 | 0.0808 |
| 15 | Household size | Gender | -0.4407 | 0.1750 | 28 | -2.5183 | 0.0118 |
| 16 | Household size | Age | -0.0111 | 0.0055 | 28 | -2.0360 | 0.0417 |
| 17 | Level of education | Age | -0.0588 | 0.0182 | 29 | -3.2242 | 0.0031 |
| 18 | Level of education | Gender | -0.1372 | 0.7186 | 29 | -0.1909 | 0.8499 |
| 19 | Residence time | Household size | -0.2093 | 1.0895 | 28 | -0.1921 | 0.8491 |
| 20 | Residence time | Age | 0.8512 | 0.1131 | 28 | 7.5250 | 0.0000 |
| 21 | Residence time | Gender | 5.1200 | 4.7736 | 28 | 1.0726 | 0.2926 |
| 22 | Professional occupation | Level of education | 0.1098 | 0.0687 | 27 | 1.5968 | 0.1220 |
| 23 | Professional occupation | Residence time | 0.0045 | 0.0116 | 27 | 0.3892 | 0.7002 |
| 24 | Professional occupation | Gender | 0.4243 | 0.2655 | 27 | 1.5979 | 0.1217 |
| 25 | Professional occupation | Age | 0.0243 | 0.0118 | 27 | 2.0602 | 0.0491 |
