# Supplementary material for: Testing the expectancy-disconfirmation theory: Geography, employment status and household size of local communities determine their perspectives of a local mine business in South Africa
Source: PLoS One. 2022 Jul 25;17(7):e0270815. doi: 10.1371/journal.pone.0270815 (PMC9312416; doi:10.1371/journal.pone.0270815)
Supplement: S5 Table — (DOC) [file pone.0270815.s005.doc]

**S5 Table:** Path coefficients for all relationships among variables included in the SEM model for the *Ramoga* community.

|  | Response | Predictor | Estimate | Std.Error | DF | Crit.Value | P.Value |
| --- | --- | --- | --- | --- | --- | --- | --- |
| 1 | Happiness | Level of education | 0.3544 | 0.2672 | 23 | 1.3265 | 0.1847 |
| 2 | Happiness | Residence time | 0.0145 | 0.0378 | 23 | 0.3834 | 0.7014 |
| 3 | Happiness | Gender | -0.1222 | 1.0049 | 23 | -0.1216 | 0.9032 |
| 4 | Happiness | Professional occupation | 0.1289 | 0.4869 | 23 | 0.2648 | 0.7912 |
| 5 | Happiness | Age | -0.0113 | 0.0408 | 23 | -0.2760 | 0.7826 |
| 6 | Happiness | Household size | 0.1538 | 0.1796 | 23 | 0.8563 | 0.3918 |
| 7 | Satisfation level | Level of education | 0.0940 | 0.2361 | 22 | 0.3980 | 0.6906 |
| 8 | Satisfation level | Residence time | 0.0149 | 0.0400 | 22 | 0.3725 | 0.7095 |
| 9 | Satisfation level | Gender | 0.4424 | 1.0156 | 22 | 0.4357 | 0.6631 |
| 10 | Satisfation level | Happiness | 2.6968 | 1.1038 | 22 | 2.4433 | 0.0146 |
| 11 | Satisfation level | Professional occupation | -0.3628 | 0.5104 | 22 | -0.7108 | 0.4772 |
| 12 | Satisfation level | Household size | -0.0469 | 0.1934 | 22 | -0.2424 | 0.8085 |
| 13 | Satisfation level | Age | 0.0144 | 0.0415 | 22 | 0.3475 | 0.7282 |
| 14 | Household size | Level of education | -0.0186 | 0.0467 | 26 | -0.3981 | 0.6905 |
| 15 | Household size | Gender | -0.1516 | 0.2317 | 26 | -0.6543 | 0.5129 |
| 16 | Household size | Age | 0.0056 | 0.0088 | 26 | 0.6325 | 0.5271 |
| 17 | Level of education | Age | -0.1087 | 0.0306 | 27 | -3.5481 | 0.0014 |
| 18 | Level of education | Gender | -1.6825 | 0.9037 | 27 | -1.8618 | 0.0736 |
| 19 | Residence time | Household size | 0.1910 | 0.9713 | 26 | 0.1966 | 0.8457 |
| 20 | Residence time | Age | 0.5039 | 0.1673 | 26 | 3.0124 | 0.0057 |
| 21 | Residence time | Gender | 6.2262 | 4.8706 | 26 | 1.2783 | 0.2124 |
| 22 | Professional occupation | Level of education | 0.0826 | 0.0829 | 25 | 0.9958 | 0.3289 |
| 23 | Professional occupation | Residence time | 0.0073 | 0.0157 | 25 | 0.4654 | 0.6457 |
| 24 | Professional occupation | Gender | -0.3250 | 0.3945 | 25 | -0.8239 | 0.4178 |
| 25 | Professional occupation | Age | 0.0080 | 0.0160 | 25 | 0.5032 | 0.6192 |
