# Supplementary material for: Testing the expectancy-disconfirmation theory: Geography, employment status and household size of local communities determine their perspectives of a local mine business in South Africa
Source: PLoS One. 2022 Jul 25;17(7):e0270815. doi: 10.1371/journal.pone.0270815 (PMC9312416; doi:10.1371/journal.pone.0270815)
Supplement: S6 Table — (DOC) [file pone.0270815.s006.doc]

**S6 Table:** Path coefficients for all relationships among variables included in the SEM model for the *Moruleng* community.

|  | Response | Predictor | Estimate | Std.Error | DF | Crit.Value | P.Value |
| --- | --- | --- | --- | --- | --- | --- | --- |
| 1 | Happiness | Level of education | -0.4059 | 0.3627 | 23 | -1.1191 | 0.2631 |
| 2 | Happiness | Residence time | 0.0426 | 0.0766 | 23 | 0.5565 | 0.5779 |
| 3 | Happiness | Gender | -1.0904 | 1.2365 | 23 | -0.8819 | 0.3778 |
| 4 | Happiness | Professional occupation | 1.5049 | 0.7558 | 23 | 1.9910 | 0.0465 |
| 5 | Happiness | Age | -0.1487 | 0.1167 | 23 | -1.2740 | 0.2027 |
| 6 | Happiness | Household size | 0.0769 | 0.2474 | 23 | 0.3110 | 0.7558 |
| 7 | Satisfation level | Level of education | 77.2008 | 29961.4598 | 22 | 0.0026 | 0.9979 |
| 8 | Satisfation level | Residence time | -37.4842 | 14104.3715 | 22 | -0.0027 | 0.9979 |
| 9 | Satisfation level | Gender | 93.1040 | 59746.1027 | 22 | 0.0016 | 0.9988 |
| 10 | Satisfation level | Happiness | 872.3561 | 322723.9290 | 22 | 0.0027 | 0.9978 |
| 11 | Satisfation level | Professional occupation | -314.4040 | 120340.1851 | 22 | -0.0026 | 0.9979 |
| 12 | Satisfation level | Household size | -22.7326 | 9712.8288 | 22 | -0.0023 | 0.9981 |
| 13 | Satisfation level | Age | 42.7963 | 16138.2717 | 22 | 0.0027 | 0.9979 |
| 14 | Household size | Level of education | 0.0241 | 0.0535 | 26 | 0.4513 | 0.6518 |
| 15 | Household size | Gender | -0.1789 | 0.1662 | 26 | -1.0765 | 0.2817 |
| 16 | Household size | Age | -0.0069 | 0.0060 | 26 | -1.1501 | 0.2501 |
| 17 | Level of education | Age | 0.0153 | 0.0199 | 27 | 0.7675 | 0.4495 |
| 18 | Level of education | Gender | -0.2416 | 0.6073 | 27 | -0.3978 | 0.6939 |
| 19 | Residence time | Household_size | 0.4192 | 0.5801 | 26 | 0.7226 | 0.4764 |
| 20 | Residence time | Age | 1.0045 | 0.0791 | 26 | 12.6963 | 0.0000 |
| 21 | Residence time | Gender | 6.2856 | 2.4167 | 26 | 2.6009 | 0.0151 |
| 22 | Professional occupation | Level of education | -0.0085 | 0.1085 | 25 | -0.0786 | 0.9379 |
| 23 | Professional occupation | Residence time | -0.0551 | 0.0282 | 25 | -1.9527 | 0.0621 |
| 24 | Professional occupation | Gender | 0.4202 | 0.3802 | 25 | 1.1050 | 0.2797 |
| 25 | Professional occupation | Age | 0.0773 | 0.0303 | 25 | 2.5516 | 0.0172 |
