# Supplementary material for: Testing the expectancy-disconfirmation theory: Geography, employment status and household size of local communities determine their perspectives of a local mine business in South Africa
Source: PLoS One. 2022 Jul 25;17(7):e0270815. doi: 10.1371/journal.pone.0270815 (PMC9312416; doi:10.1371/journal.pone.0270815)
Supplement: S7 Table — (DOC) [file pone.0270815.s007.doc]

**S7 Table:** Path coefficients for all relationships among variables included in the SEM model for the *Manamakgoteng* community.

|  | Response | Predictor | Estimate | Std.Error | DF | Crit.Value | P. Value |
| --- | --- | --- | --- | --- | --- | --- | --- |
| 1 | Happiness | Level of education | 0.2151 | 0.1724 | 27 | 1.2481 | 0.2120 |
| 2 | Happiness | Residence time | -0.1194 | 0.0655 | 27 | -1.8227 | 0.0683 |
| 3 | Happiness | Gender | 0.4088 | 0.9386 | 27 | 0.4356 | 0.6632 |
| 4 | Happiness | Professional occupation | -1.3211 | 0.6600 | 27 | -2.0018 | 0.0453 |
| 5 | Happiness | Age | 0.1479 | 0.0729 | 27 | 2.0286 | 0.0425 |
| 6 | Happiness | Household size | -0.1584 | 0.2723 | 27 | -0.5817 | 0.5608 |
| 7 | Satisfation level | Level of education | 0.0699 | 0.1700 | 26 | 0.4113 | 0.6809 |
| 8 | Satisfation level | Residence time | -0.0403 | 0.0611 | 26 | -0.6592 | 0.5097 |
| 9 | Satisfation level | Gender | 1.2067 | 1.0204 | 26 | 1.1826 | 0.2370 |
| 10 | Satisfation level | Happiness | 2.3302 | 1.0574 | 26 | 2.2038 | 0.0275 |
| 11 | Satisfation level | Professional occupation | -0.0652 | 0.6224 | 26 | -0.1048 | 0.9166 |
| 12 | Satisfation level | Household size | 0.3553 | 0.2786 | 26 | 1.2754 | 0.2022 |
| 13 | Satisfation level | Age | 0.0626 | 0.0700 | 26 | 0.8935 | 0.3716 |
| 14 | Household size | Level of education | -0.0371 | 0.0249 | 30 | -1.4904 | 0.1361 |
| 15 | Household size | Gender | -0.1732 | 0.1468 | 30 | -1.1796 | 0.2382 |
| 16 | Household size | Age | -0.0072 | 0.0049 | 30 | -1.4783 | 0.1393 |
| 17 | Level of education | Age | -0.0742 | 0.0304 | 31 | -2.4389 | 0.0207 |
| 18 | Level of education | Gender | -0.3098 | 1.0232 | 31 | -0.3028 | 0.7641 |
| 19 | Residence time | Household size | -1.4090 | 0.8729 | 30 | -1.6142 | 0.1170 |
| 20 | Residence time | Age | 0.9435 | 0.0961 | 30 | 9.8208 | 0.0000 |
| 21 | Residence time | Gender | 1.6599 | 3.2544 | 30 | 0.5100 | 0.6138 |
| 22 | Professional occupation | Level of education | 0.0088 | 0.0497 | 29 | 0.1770 | 0.8608 |
| 23 | Professional occupation | Residence time | 0.0128 | 0.0157 | 29 | 0.8136 | 0.4225 |
| 24 | Professional occupation | Gender | 0.4365 | 0.2873 | 29 | 1.5196 | 0.1394 |
| 25 | Professional occupation | Age | 0.0089 | 0.0180 | 29 | 0.4942 | 0.6249 |
